# Decoding the role of H19 in cholestatic liver injury using snRNA-seq, spatial transcriptomics, and machine learning-based disease prediction

**DOI:** 10.1186/s13578-026-01590-3

**Published:** 2026-05-29

**Authors:** Grayson W. Way, Xixian Jiang, Hongkun Lu, Nan Wu, Derrick Zhao, Yun-ling Tai, Sareh Bayatpour, Xuan Wang, Huiping Zhou

**Affiliations:** 1 Department of Microbiology and Immunology, Richmond VA Medical Center, Virginia Commonwealth University, 1220 East Broad Street, MMRB-5044, Richmond, VA 23298-0678 USA; 2 Richmond Veterans Affairs Medical Center, Richmond, VA 23249 USA

**Keywords:** Primary sclerosing cholangitis, Cholestasis, SPP1, Machine learning, H19, Long non-coding RNA

## Abstract

**Background:**

Despite recent advances, Primary Sclerosing Cholangitis (PSC)-a chronic obstructive biliary disease-still lacks effective therapies to prevent disease progression or the need for liver transplantation. Moreover, up to 30% of transplant recipients experience recurrence. Long non-coding RNA H19 (H19) has been implicated in promoting PSC progression, yet the cellular and molecular mechanisms underlying its pathogenic role remain incompletely understood.

**Results:**

Liver tissues from age- and sex-matched wild type (WT), H19 knockout (H19KO), Mdr2 knockout (Mdr2KO), and double-knockout (DKO; Mdr2KO/H19KO) mice were analyzed using single-nucleus RNA sequencing (snRNAseq) and GeoMx spatial transcriptomics to define the cell type and spatially specific effects of H19 deletion in cholestatic liver injury. Machine learning models were built to develop cell-type-specific gene prediction signatures and cross-validated using the human dataset GSE243981. A disease-associated cholangiocyte subcluster that increased in Mdr2KO but was markedly reduced in DKO mice was identified. SPP1 signaling was significantly dysregulated in cholestatic liver injury and mitigated following H19 deletion. Translationally conserved healthy (Clu, Spp1) and diseased (Csmd1, Slco3a1, Cftr) cholangiocyte markers were identified. When validated in a human patient dataset (GSE243981), our machine-learning prediction models achieved AUC values > 0.869. Finally, spatial analyses demonstrated that the mitigation of disease-associated gene expression following H19 deletion was specifically restricted to hepatocytes within the bile duct region.

**Conclusions:**

H19 deletion mitigates cholestatic injury by suppressing pathogenic cholangiocyte states, normalizing Spp1-mediated signaling, and shifting transcriptional programs specifically within the periductal niche. Furthermore, our machine-learning signatures demonstrate robust cross-species translation and may benefit future post-transplant analytics and disease recurrence predictions.

**Supplementary Information:**

The online version contains supplementary material available at 10.1186/s13578-026-01590-3.

## Introduction

Primary sclerosing cholangitis (PSC) is a chronic, progressive hepatobiliary disease characterized by biliary fibrosis, obstructive cholestasis, and inflammation of the intrahepatic and extrahepatic bile ducts [1–3]. The disruption of bile acid homeostasis contributes to a cascade of metabolic and immune dysregulation, as bile acids are important signaling molecules. PSC progresses along a spectrum with eventual development of end-stage liver failure [4]. PSC is also associated with a markedly increased risk of malignancies, such as cholangiocarcinoma, gallbladder carcinoma, hepatocellular carcinoma, and colorectal carcinoma [5–10]. In the United States, PSC is the fifth leading indication for liver transplantation [11]. Furthermore, recurrence occurs in up to 30% of transplant recipients [11]. Despite the severity of the disease, no proven pharmacotherapies exist, largely due to an incomplete understanding of the cellular and molecular mechanisms underlying PSC pathogenesis [11].

The long non-coding RNA H19 (H19) has emerged as an important regulator of PSC disease progression [12, 13]. Previous research in our lab and others has shown that H19, which is normally not expressed in healthy hepatic tissues, is upregulated in PSC and exacerbates disease progression in Mdr2KO mice (the gold standard mouse model for PSC) [12, 14–19]. Elucidating the mechanisms by which H19 deletion leads to transcriptional network and cellular function alterations is key to uncovering novel therapeutic targets. To achieve this, we leveraged single-nucleus RNA sequencing (snRNA-seq) and spatial transcriptomic profiling. While single-cell RNA sequencing (scRNAseq) has advantages for profiling immune cells, snRNAseq offers several distinct benefits, including compatibility with frozen tissue, reduced dissociation-induced artifacts, and superior accuracy in profiling hepatocytes and cholangiocytes [20]. Spatial transcriptomic technology, such as GeoMx^®^ Digital Spatial Profiler (DSP), allows transcriptomic analysis at specific regions based on histological morphology.

In our previous work, we demonstrated that genetic deletion of H19 significantly reduced liver fibrosis and slowed disease progression in Mdr2KO mice [15, 17]. To investigate the cell-type-specific and spatial transcriptomic alterations induced by H19-deletion, we performed snRNAseq and NanoString GeoMx DSP using the mouse Whole Transcriptome Atlas (WTA) panel with liver tissues from wild type (WT), Mdr2KO, H19KO, and Mdr2KO/H19KO (DKO) mice. To extend the translational relevance of our findings, we integrated publicly available scRNAseq and snRNAseq datasets from human patients with cholestatic liver diseases and applied machine learning approaches to identify disease-associated gene signatures to predict disease progression.

## Materials and methods

### Animal studies

C57BL/6J Mdr2KO mice were gifted by Dr. Daniel Goldenberg, Department of Pathology, Hadassah-Hebrew University Medical Center, Jerusalem, Israel. Maternal C57BL/6J H19^ΔExon1/+^ (H19KO) mice were generated and gifted by Dr. Karl Pfeifer at the NIH. Dr. Jian-Ying Wang at the University of Maryland (Baltimore, MD, USA) provided the H19KO mice. Mdr2KO and H19KO (DKO) mice were generated as previously described [17]. The gender and age-matched littermates of WT, H19KO, Mdr2KO and DKO (female, 6-month-old) were used. Mice were housed under 12 h/12 h light and dark cycle with unrestricted access to water and standard chow *ad libitum*. All animal experiments followed institutional guidelines for ethical animal studies and were approved by the VCU Institutional Animal Care and Use Committee.

For GeoMx spatial transcriptomics, eight liver samples were collected from 6-month-old female mice (four Mdr2⁻/⁻ and four DKO). For single-nucleus RNA sequencing (snRNA-seq), liver tissues from 6-month-old female mice (one per genotype: WT, H19KO, Mdr2KO, and DKO) were flash-frozen in liquid nitrogen and stored at −80 °C until processing. To validate our key snRNA-seq findings and overcome the limitation of a single biological replicate for sequencing, independent qPCR validation was performed on a larger cohort of mice (n = 5–6 per group) to confirm the expression changes of key cluster markers. Region-of-interest (ROI) selection of GeoMx was guided by three fluorescent morphology markers-AF488-conjugated anti-αsmooth muscle actin (αSMA), AF594-conjugated anti-CD68, and AF647-conjugated anti-EpCAM-along with the nuclear dye Syto 83, enabling identification of hepatocyte-, macrophage-, and bile duct-containing regions. To eliminate potential selection bias, the region selector was blinded to the tissue samples’ genotype origin during ROI selection. Regions were strictly defined based on quantitative proximity to bile ducts: ‘close proximity’ was defined as hepatocytes within an approximately 400 µm radius centered on the adjacent bile duct, while ‘isolated’ hepatocytes were defined as those located outside of this 400µm radius. Tissue preparation and processing were performed following the manufacturer’s protocol.

## scRNAseq and snRNAseq data analysis

scRNAseq and snRNAseq analyses of both our dataset and the GSE243981 dataset were performed in R (version 4.3.3) utilizing the R package Seurat (version 5.1.0) [21, 22]. For our mouse snRNA-seq data, nuclei with > 2.5% mitochondrial gene content were excluded. For GSE243981, mitochondrial content filters of 5% (snRNA-seq) and 25% (scRNA-seq) were applied. Data normalization and scaling were performed using Seurat’s SCTransform function and data integration was performed utilizing Harmony, accounting for known batch variables (i.e., different experimental techniques used: scRNAseq, snRNAseq, 3’ and 5’ sequencing) following the Seurat SCTransform workflow [23]. Predicted cell-cell communication were inferred using the R package CellChatv2 (version 2.1.0) [24]. Pseudotime analyses for cell type progression with disease was performed using Monocle3 (version 1.4.26) [25]. LASSO regression analysis was performed using the R package glmnet (version 4.1.10) [26, 27]. Data visualization and statistical analyses were performed with SeuratExtend (version 1.2.5) [28]. Statistical significance was determined using the MAST algorithm via Seurat’s FindMarkers function.

## Machine learning modeling methods

To assess the robustness and predictive accuracy of input features, four supervised machine learning models were developed and evaluated: a multilayer perceptron (MLP) neural network, an extreme gradient boosting (XGBoost) classifier, a random forest classifier, and a logistic regression model. For initial exploratory validation, models were trained and tested on mouse data using a 70/30 train–test split. For disease prediction, WT and H19KO mice were grouped versus Mdr2KO and DKO, while for H19 prediction, WT and Mdr2KO were grouped versus H19KO and DKO. To avoid potential data leakage artifacts inherent to train-test splits within our specific mouse cohort, we prioritized cross-species validation to determine the true generalizability of our disease prediction models. For cross-species validation, each model was trained on the complete mouse dataset and subsequently tested on the entire human dataset (GSE243981). Mouse gene symbols were converted to their human orthologs using the gorth function in the gprofiler2 R package to enable translational comparison [29]. All models were evaluated using the area under the receiver operating characteristic curve (ROC-AUC) as the primary performance metric with the R package pROC [30]. For the human dataset cross-validation, binary health status ground-truth labels were explicitly defined as “Healthy” (0) for Neurologically Deceased Donor (NDD) samples, and “Diseased” (1) for both PSC and PBC patient samples.

## GeoMx data analysis

GeoMx^®^ data analysis was performed using the StandR R package following their pipeline [31]. QC was performed at both the region of interest (ROI) level and gene level to remove low-quality data. ROIs were filtered based on nuclear count, surface area, and library size using default thresholds. PCA was performed using the runPCA function from the scater package (version 1.36.0), and clustering was visualized using Uniform Manifold Approximation and Projection (UMAP) [32]. Different normalization (TMM, CPM, Upper quartile) and batch correction methods (RUV4, LimmaRemoveBatch, SVA) were tested. Ultimately, TMM normalization and RUV4 batch correction were used based on PCA and cluster separation statistics. Differential gene expression (DGE) analysis was performed using the limma R package (version 3.64.3) [33]. Significance was also tested and confirmed using DESeq2 (Version 1.48.1) [34].

## Results

### Identification of a disease-specific cholangiocyte cluster

**Fig. 1:**
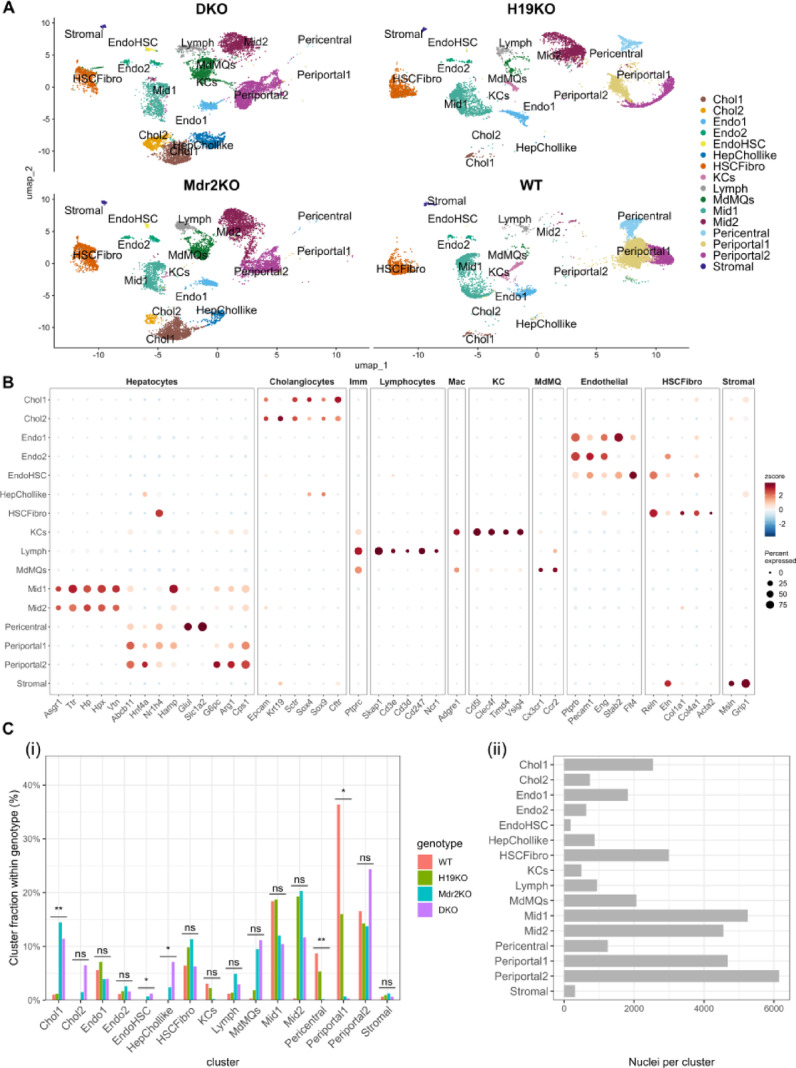
Single-nucleus transcriptomic atlas of healthy and cholestatic mouse livers. **A** Uniform Manifold Approximation and Projection (UMAP) visualization of the clustering of 35,488 nuclei sequenced from all samples, WT, H19KO, Mdr2KO and DKO, respectively). **B** Dot plot showing canonical marker genes used to assign cell-type identities to each cluster. **C** Bar plots depicting (i) the proportional representation of each cluster by genotype and (ii) the absolute nuclei counts for each cell type (Significance determined using Welch’s t-test comparing WT and H19KO percentages vs. Mdr2KO and DKO, * *p* < 0.05). Note: Mice only contained one mouse per-genotype. Primarily used for exploratory purposes

**Fig. 2:**
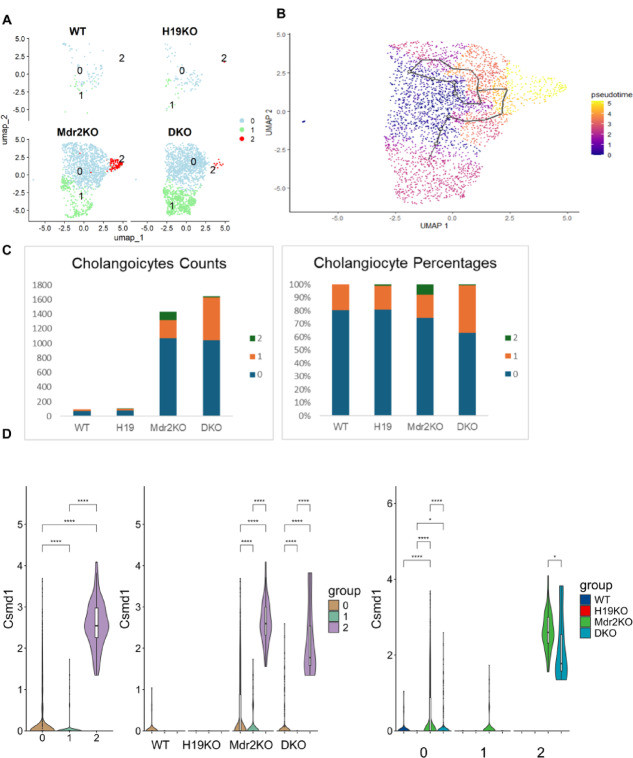
snRNAseq reveals disease-associated cholangiocyte subtype.** A** UMAP visualization of subclustered cholangiocytes across all genotypes,** B** Pseudotime trajectory of cholangiocyte subclusters, with color denoting progression along the disease axis from healthy (purple) to late-stage disease (yellow), **C** Bar plots showing the proportional distribution of cholangiocyte subtypes across samples, **D** Violin plots illustrating *Csmd1* expression—the top marker of cluster 2—displayed (left) across all clusters, (middle) across clusters within each genotype, and (right) by cluster stratified by genotype. (*p < 0.05, **p < 0.01, ***p < 0.001, ****p < 0.0001; Wilcoxon test implemented *via* Seurat Extend). Note: Mice only contained one mouse per-genotype. Primarily used for exploratory purposes

Initial clustering and cell type annotation of the snRNAseq data identified 16 distinct clusters from 35,488 nuclei across all four genotypes. UMAP visualization confirmed proper integration across all genotypes and experimental techniques (Fig. 1A). Cell identity was assigned based on the expression of canonical maker genes, demonstrating high-fidelity clustering (Fig. 1B). Compared to WT and H19KO, Mdr2KO and DKO mice exhibited drastic increases in cholangiocytes, cholangiocyte-like hepatocytes, lymphocytes, and monocyte-derived macrophages (MdMQs) in terms of numbers and percent cell composition, accompanied by a reduction in hepatocytes and Kupffer cells, compared to their healthy counterparts (cholangiocytes, cholangiocyte-like hepatocytes, and hepatocytes percentage of cells *p* < 0.05) (Fig. 1C). Subclustering of cholangiocytes identified a disease-associated cholangiocyte cluster (cluster 2) (Fig. 2A). Pseudotime trajectory analysis using monocle3 indicated that Cluster 2 corresponded to a late-stage disease state (Fig. 2B). Notably, H19 deletion in Mdr2KO mice significantly reduced both the number and percentage of cholangiocytes within this cluster (Fig. 2C). The top marker gene for cluster 2 was *Csmd1* (CUB And Sushi Multiple Domains 1), which was markedly upregulated in disease and downregulated upon H19 deletion (Supp. Figs. 1 and 2D).

## Cellular pathways altered in PSC and ameliorated by H19 deletion

**Fig. 3:**
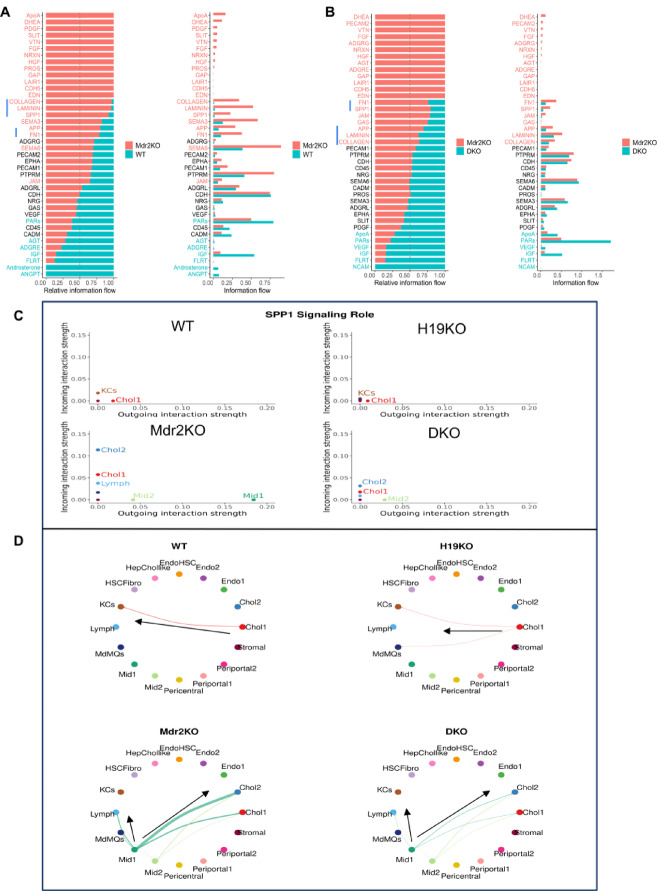
H19 deletion significantly ameliorates disease-associated cell-signaling alterations. Relative information flow plots showing pathways significantly altered between Mdr2KO and WT (**A**) or DKO (**B**) mice. Colored pathways denote significant changes (peach = increased, teal = decreased in Mdr2KO). Scatter (**C**)and circle (**D**) plots illustrating SPP1 signaling dynamics. In healthy mice, Spp1 is primarily expressed by cholangiocytes, but shifts to mid-lobular hepatocytes in Mdr2KO livers; H19 deletion markedly reduces this ectopic hepatocyte expression. Line color indicates ligand source, and line width represents interaction strength. Note: Mice only contained one mouse per-genotype. Primarily used for exploratory purposes

**Fig. 4:**
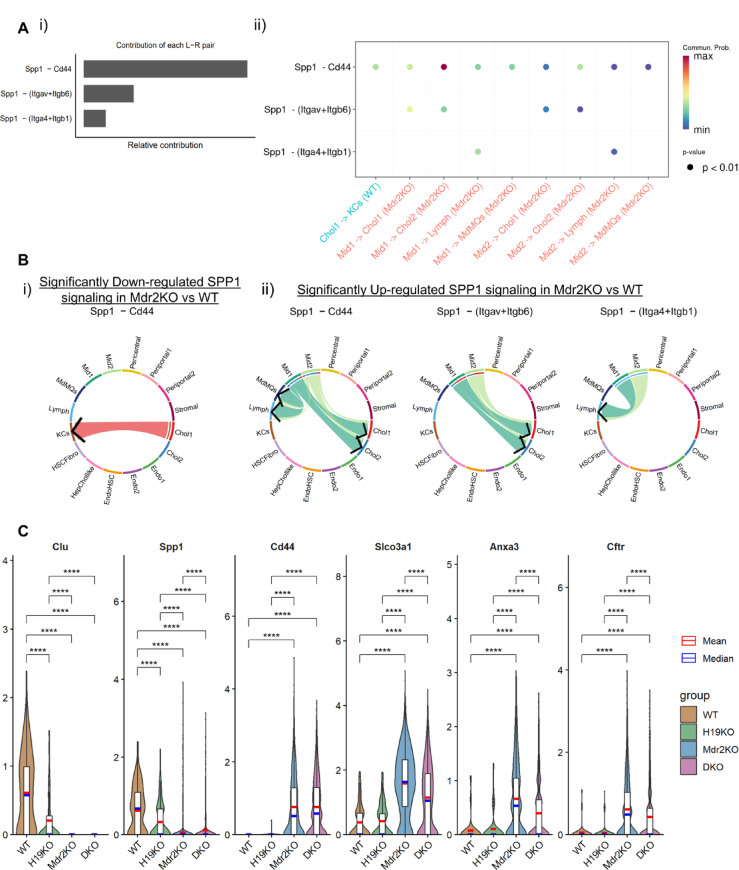
Altered signaling pathways and genes distinguishing cholangiocyte disease status. **A** (i) Relative contribution of the SPP1 signaling pathway of Mdr2KO mice. (ii) Net-signaling role dot plot showing predicted SPP1 ligand-receptor interactions; dot color indicates communication probability (brown = maximum, blue = minimum). Blue and peach labels denote interactions enriched in WT and Mdr2KO, respectively. **B** Chord plots depicting cell-to-cell communication for each predicted ligand-receptor pair, separated by WT (i) and Mdr2KO (ii). Line color indicates ligand source; line thickness reflects interaction strength. Differential expression and LASSO analyses identified six genes for disease-prediction modeling. **C** Violin plots showing expression of these genes in mice. H19 deletion significantly reversed expression of disease-associated genes (*Slco3a1*, *Anxa3*, *Cftr*, *Cd44*) toward WT levels, except *Cd44*. (*p < 0.05, **p < 0.01, ***p < 0.001, ****p < 0.0001; Wilcoxon test, SeuratExtend). Note: Mice only contained one mouse per-genotype. Primarily used for exploratory purposes

CellChatv2 analysis was performed to identify significant cell-cell signaling networks and crosstalk interactions altered in Mdr2KO mice and to evaluate pathways modulated by H19 deletion. The total number of inferred interactions identified for WT, H19KO, Mdr2KO, and DKO were 255, 180, 573, and 438, respectively (Supp. Fig. 2). Importantly, we opted not to artificially downsample the data to equalize cell proportions across genotypes prior to this analysis. A key feature of cholestatic disease progression is the shift in tissue cellular composition—driven by immune cell recruitment, cell death, and state transitions such as the ductular reaction. Because these compositional changes directly alter the probability of cell-cell contact and ligand-receptor engagement in vivo, retaining the true cell proportions preserves these biologically meaningful microenvironmental signals. However, because these interaction counts are derived from single biological replicates (*n* = 1 per genotype), the overall interaction numbers should be interpreted with caution as exploratory findings. Among the pathways examined, we focused specifically on Spp1 (osteopontin), collagen, laminin, and FN1 (fibronectin) signaling, as they are key components of the extracellular matrix (ECM) closely tied to the fibrotic pathology of PSC. While Spp1 was selected due to its known association with cholangiocytes, a primary cell type of interest in cholestatic injury. These pathways were all significantly upregulated in Mdr2KO compared to WT and were normalized toward WT in DKO mice (*p*-value < 0.05) (Fig. 3A-B, Supp. Figs. 3, 4 and 5). Notably, Spp1 emerged as a major signaling pathway dysregulated in cholestatic liver injury and restored by H19 deletion. In healthy liver, *Spp1* expression is predominately observed in cholangiocytes, mediating signaling to Kupffer cells. In contrast, during disease progression, Spp1 expression shifts to mid-lobular hepatocytes, indicating altered paracrine signaling (Fig. 3C-D, Supp. Fig. 6A-B). H19 deletion significantly reduces Spp1 expression and outgoing Spp1-mediated signaling from mid-lobular hepatocytes (*p* < 0.05) (Fig. 3C-D). Ligand-receptor analysis revealed that the top predicted interactions in the Spp1 pathway were Spp1-Cd44, Spp1-(Itgav + Itgb6), and Spp1-(Itga4 + Itgb1). Notably, Spp1-Cd44 signaling between cholangiocytes and Kupffer cells was present only in healthy liver and was lost in Mdr2KO mice, whereas the other Spp1 interactions were uniquely predicted in Mdr2KO mice (Fig 4 A-B).

## Diseased cholangiocyte gene prediction model and identification of novel healthy markers

Differential gene expression (DGE) analysis of subclustered cholangiocytes, combined with LASSO regression-based feature selection, identified six genes (*Clu*, *Spp1*, *Slco3a1*, *Cd44*, *Anxa3*, *Cftr*) for machine learning-based prediction modeling of cholangiocyte disease status (Fig. 4C). Among these, *Clu* and *Spp1* were strongly associated with healthy cholangiocytes, whereas *Slco3a1*, *Cd44*, *Anxa3*, *Cftr* were enriched in diseased cholangiocytes in Mdr2KO and DKO mice (adj. *p* < 0.001) (Fig. 4C). Biologically, the emergence of this signature aligns with known cholestatic injury responses; for example, the upregulation of ion and anion transporters like *Cftr* and *Slco3a1* likely reflects a compensatory mechanism by cholangiocytes to aid in fluid secretion and bile efflux during obstructive injury. Three of the disease associated genes (*Slco3a1*, *Anxa3*, *Cftr*) were significantly reduced towards WT levels in DKO mice, indicating that H19 deletion mitigates their dysregulation (Fig. 4C). A striking spatial shift was observed for *Spp1* and *Clu*, which transitioned from cholangiocyte-restricted expression in healthy liver to ectopic expression in mid-lobular hepatocytes in diseased liver, suggesting substantial rewiring of cellular identity and intercellular signaling during disease progression (Supp. Fig. 6A–B). Cholangiocyte gene changes were confirmed in mouse and human liver tissue with real-time quantitative polymerase chain reaction (RT-qPCR) (Supp. Fig. 7).

### Cholangiocyte disease prediction model testing in humans

**Fig. 5:**
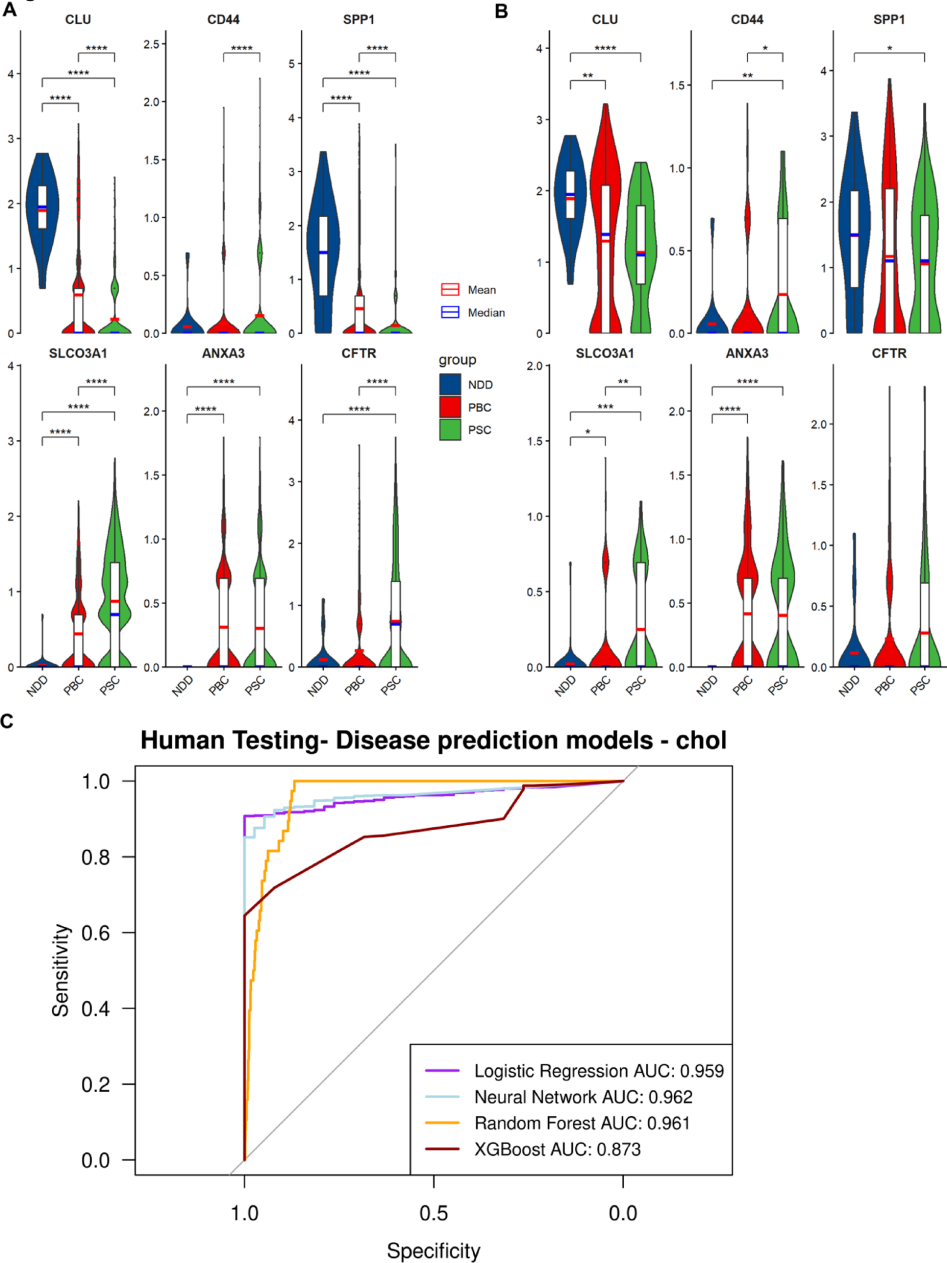
Cross-species validation of cholangiocyte disease-prediction genes. Mouse-derived disease prediction model genes (WT and H19KO versus Mdr2KO and DKO) were tested on public human cholangiocyte data (GSE243981) subclustered from sc/snRNA-seq samples integrated with Harmony to correct for sequencing-method batch effects. **A** Violin plots showing expression of prediction-model genes across neurologically deceased donors (NDD), primary biliary cholangitis (PBC), and primary sclerosing cholangitis (PSC) patients using combined sc/snRNA-seq data. **B** Violin plots showing expression using only scRNA-seq samples (*p < 0.05, **p < 0.01, ***p < 0.001, ****p < 0.0001; Wilcoxon test, SeuratExtend). **C** ROC–AUC analysis showing machine-learning model performance in distinguishing healthy versus diseased cholangiocytes in human patients. Human data testing set contained both snRNAseq and scRNAseq samples

The expression patterns of six model genes (*Clu*, *Spp1*, *Slco3a1*, *Cd44*, *Anxa3*, *Cftr*) were evaluated in human cholangiocytes using the GSE243981 dataset (both scRNAseq and snRNAseq). To evaluate disease specificity, we compared expression trends among neurologically deceased donors (NDD, healthy controls), PSC, and PBC patients. Five of the six genes demonstrated the same significant expression trends in PSC patient cholangiocytes as observed in mice (p < 0.05). Importantly, these genes largely functioned as shared ‘cholestasis-associated’ markers, exhibiting similar directional trends in both PSC and PBC patients relative to healthy NDD controls, with two notable exceptions. *Cftr* and *Cd44* were specifically increased in PSC patients when combining scRNA-seq and snRNA-seq datasets (Fig. 5). *Cftr* expression was significantly increased in the combined (sc/snRNA-seq) analysis (Fig. 5A) but did not reach significance in the scRNA-seq-only analysis (Fig. 5B). *Cd44* was consistently and significantly elevated in PSC versus PBC patients; however, it was only significantly higher relative to healthy controls in the scRNA-seq-only dataset (Fig. 5B). These six genes were subsequently used to construct cholangiocyte disease prediction models using four supervised machine learning algorithms: multilayer perceptron (MLP) neural network, XGBoost, random forest, and logistic regression. Applying our binary classification strategy (NDD as health control versus both PSC ad PBC), ROC–AUC analysis demonstrated robust performance, with AUC values > 0.873 in human cholangiocytes from GSE243981. This supports the translational relevance of the gene signatures and the predictive models across species (Fig. 5C).

### Diseased hepatocyte gene prediction model

**Fig. 6:**
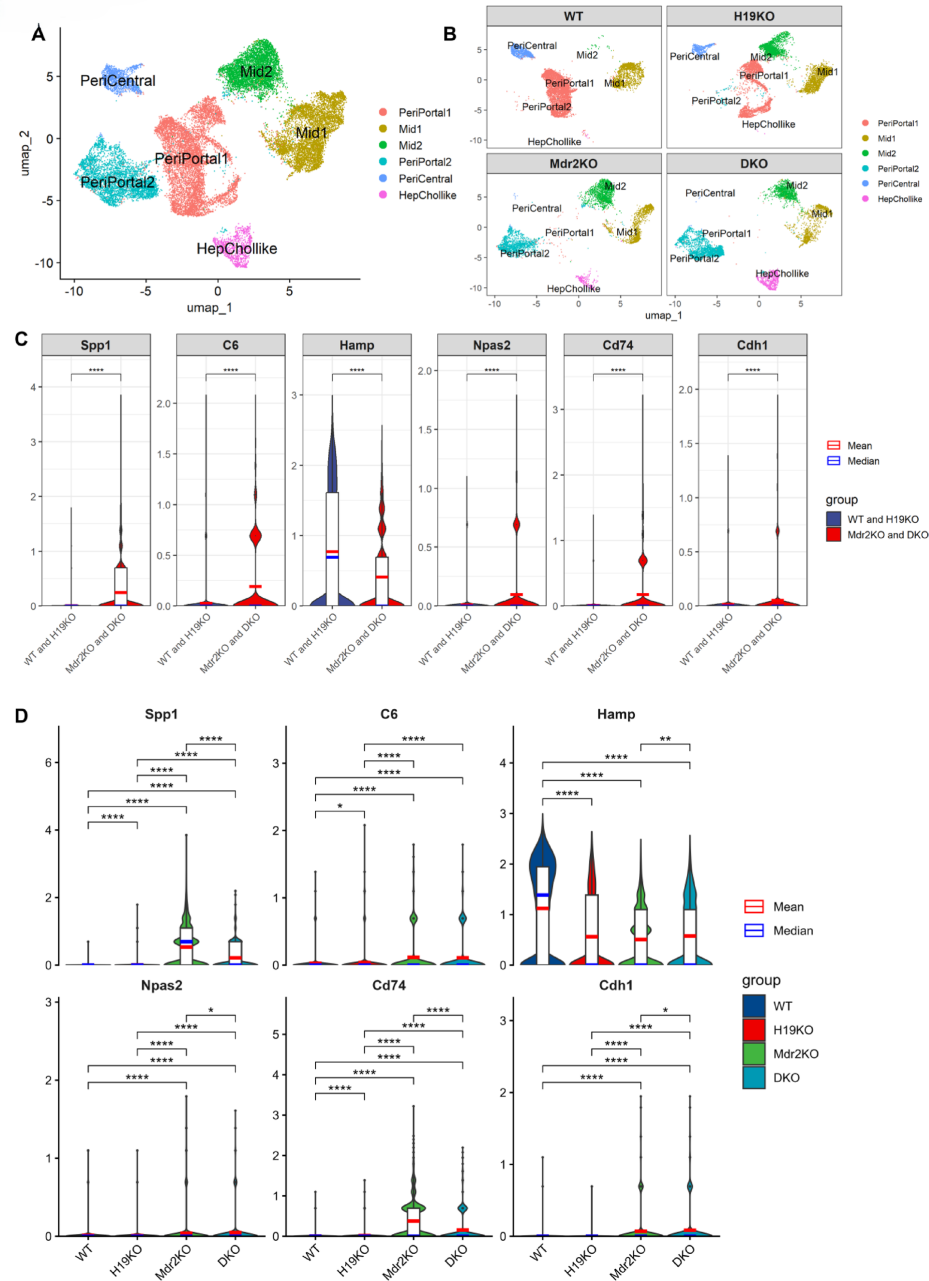
Hepatocyte disease status distinguishing genes. **A** UMAP of mouse snRNAseq subclustered hepatocytes with all samples combined and **B** split by genotype. **C** Violin plots showing significantly different expression of each gene in mice comparing WT and H19KO mice versus Mdr2KO and DKO mice. (D) Violin plots of hepatocyte disease prediction modeling genes split by genotype (*p < 0.05, **p < 0.01, ***p < 0.001, ****p < 0.0001, significance determined by Wilcoxon test using SeuratExtend)

Hepatocytes were subclustered, zonally differentiated, and UMAPs generated to visualize proper data clustering and integration (Fig. 6A-B, Supp. Fig. 8) [35, 36]. Differential gene expression combined with LASSO regression identified six translationally conserved genes: *Spp1*,* C6*,* Cdh1*,* Npas2*, *Cd74*,* and Hamp*, for the construction of a hepatocyte disease prediction model. In Mdr2KO hepatocytes, *Spp1*,* C6*,* Npas2*, *Cdh1*, and *Cd74* were all significantly upregulated, while *Hamp* was significantly reduced relative to WT (adj. *p* < 0.001) (Fig. 6C). In DKO mice, *Spp1*, and *Cd74* were significantly decreased and *Hamp* significantly increased toward WT levels (adj. *p* < 0.01), indicating that *H19* deletion mitigates hepatocyte dysregulation (Fig. 6D). CellChat v2 pathway analysis further revealed that APP signaling was significantly reduced in DKO mice (Fig. 2). Across all genotypes, the dominant ligand–receptor interaction was App–Cd74, with mid-lobular hepatocytes showing increased CD74 expression and receiving App-derived signaling in disease states (Supp. Fig. 9). Hepatocyte gene changes were confirmed in mouse and human liver tissue with RT-qPCR (Supp. Fig. 7).

### Hepatocyte disease prediction model testing in humans

**Fig. 7:**
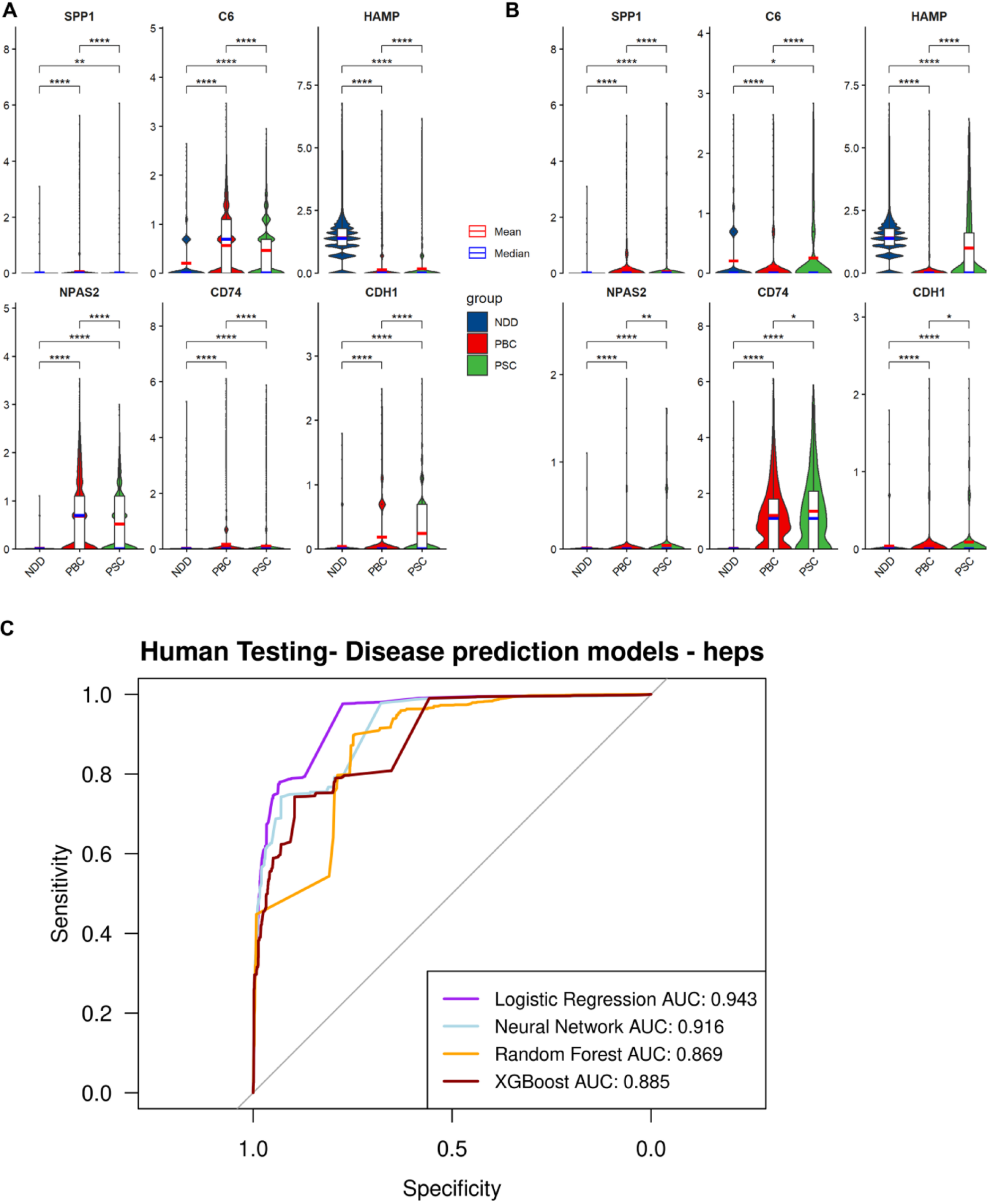
Cross-species validation of hepatocyte disease-prediction genes. Testing of hepatocyte disease prediction model genes (WT and H19KO versus Mdr2KO and DKO) derived from mouse snRNAseq data on public human dataset GSE243981. Data was integrated using Harmony for known batch variables including RNA sequencing methods (scRNAseq and snRNAseq). **A** Violin plots showing expression levels of hepatocyte disease prediction modeling genes in neurologically deceased donors (NDD), primary biliary cholangitis (PBC), and primary sclerosing cholangitis (PSC) patients combining snRNAseq and scRNAseq data. **B** Violin plots depicting expression of hepatocyte prediction genes only using scRNAseq samples (*p < 0.05, **p < 0.01, ***p < 0.001, ****p < 0.0001, significance determined by Wilcoxon test using SeuratExtend). **C** ROC-AUC analysis depicting machine learning models (MLMs) effectiveness in distinguishing healthy and diseased hepatocytes in human patients. Human data testing set contained both snRNAseq and scRNAseq samples

Original model gene selection identified *Gm13775* as a top performer in model prediction, but no known human homolog exists and was dropped due to lack of translatability (Supp. Fig. 10). The six genes used in the hepatocyte disease prediction model (Hamp, Spp1, C6, Cdh1, Npas2, and Cd74**)** demonstrated strong translational concordance in human hepatocytes from the GSE243981 dataset. To evaluate disease specificity, we compared expression trends among NDD, PSC, and PBC patients. All six genes showed consistent and significant expression changes in both the combined scRNAseq/snRNAseq dataset (Fig. 7A) and in the scRNAseq subset al.one (Fig. 7B). Importantly, these genes functioned primarily as shared cholestasis-associated markers, exhibiting similar directional changes in both PSC and PBC patients compared to NDD controls. When tested across four supervised machine learning algorithms, applying our binary classification strategy where NDD samples were labeled as healthy (0) and both PSC and PBC samples were grouped as diseased [1], these six genes achieved AUC values > 0.869 in public human datasets, confirming their cross-species robustness and strong predictive performance (Fig. 7C).

### Deletion of H19 causes cell-type specific alterations in gene expression

Random forest modeling was performed across multiple cell types to identify gene expression patterns associated with *H19* deletion. In hepatocytes, an 11 gene model (*Gphn*, *Fga*, *Malat1*, *Ahsg*, *Apob*, *Ces1c*, *Spp1*, *Cers6*, *Cd74*, *Srsf1*, and *Hspa2*) achieved an AUC score of 0.909 for distinguishing H19 deletion from WT mice (Supp. Fig. 11). In cholangiocytes, an 11-gene model (*Csmd1*,* Chka*,* Frmd4b*,* Gnas*,* Npas2*,* Agmo*,* Ly6e*,* Gbp9*,* Sorbs3*,* Usp2*, and *1600012H06Rik*) achieved an AUC score of 0.867. In macrophages, a 10 gene model (*Mat1a*,* Gm42418*,* Eef1a1*,* Rpl13a*,* Gm19951*,* Clec2d*,* Wwc1*,* Rnf128*,* Ugt2b5*, and *Tnfrsf12a*) generated an AUC score 0.764. Importantly, several genes within these models overlapped with those reversed by H19 deletion-that is, genes upregulated (or downregulated) in Mdr2KO vs. WT and reversed toward WT levels in DKO mice, indicating mitigation of PSC-associated dysregulation. Notable examples include *Spp1* and *Cd74* in hepatocytes, and *Frmd4b*, *Gnas*, *Chka*, *Csmd1*, and *Agmo* in cholangiocytes (Supp. Tables 1 and 2).

### Spatial transcriptomics reveal that H19 deletion-mediated amelioration in hepatocytes is restricted to hepatocytes in close proximity to bile ducts

**Fig. 8:**
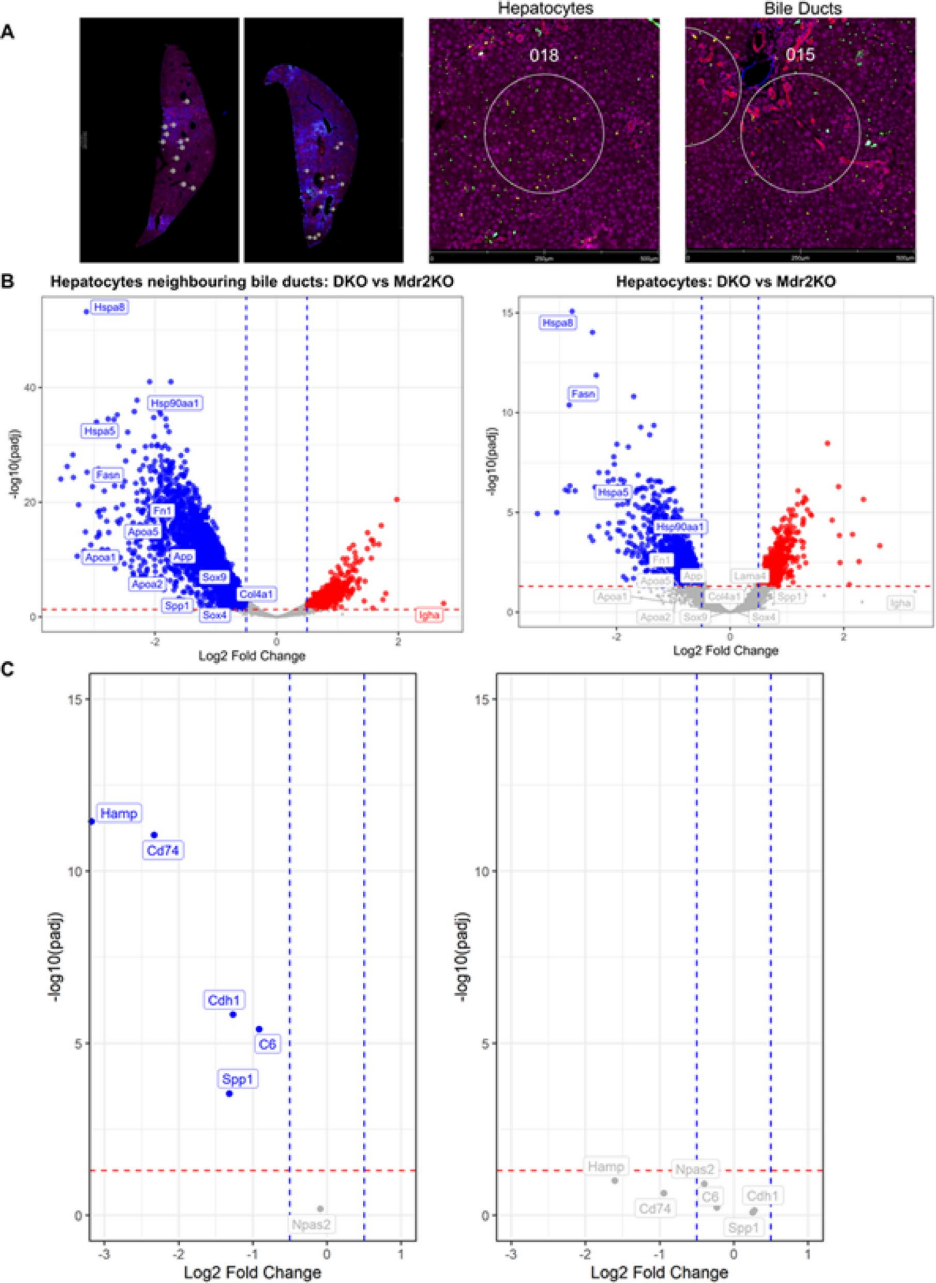
GeoMx spatial transcriptomics reveals H19 deletion’s amelioration of disease associated gene expression is spatially restricted. **A** Representative immunofluorescence images illustrating GeoMx DSP region-of-interest (ROI) selection. **B** Volcano plots showing genes significantly altered by H19 deletion in Mdr2KO mice within hepatocytes adjacent to bile ducts (left) or isolated hepatocytes (right). **C** Volcano plots showing differential expression of hepatocyte disease-prediction genes depending on proximity to bile ducts (left) versus non-adjacent regions (right). Significance thresholds: adjusted *p* < 0.05 and |log_2_C| > 0.5, determined by both DESeq2 and StandR limma-voom analyses with RUV4 batch correction;* p*-values were adjusted using the Benjamini–Hochberg method

Spatial transcriptomics was utilized to investigate spatially defined effects of H19 deletion on hepatic gene expression. ROIs were selected to compare differences in isolated hepatocytes and hepatocytes in close proximity to bile ducts (within approximately 400 μm radius centered on the bile duct) (Fig. 8A). DGE analysis of the different ROIs comparing DKO vs. Mdr2KO mice yielded several common and unique significant DEGs (Fig. 8B). Common genes decreased in DKO mice vs. Mdr2KO across both regions were genes associated with cellular stress responses, such as *Hspa5*, *Hspa8*, and *Hsp90aa1* (*p* < 0.01 across both regions), and fatty acid synthesis gene *Fasn* (*p* < 0.01); however, several cholesterol metabolism genes were only significantly reduced in hepatocytes neighboring bile ducts (e.g., *Apoa1*, *Apoa2*, and *Apoa5*) (Fig. 8B). Several genes related to significant disease-associated pathways (APP, Collagen, FN1, LAMININ) mitigated following H19 deletion, as well as hepatocyte-to-cholangiocyte transition marker genes (*Sox4* and *Sox9*), are only significantly reduced in regions with hepatocytes neighboring bile ducts (Fig. 8B). Four of the six hepatocyte disease-associated prediction modeling genes were only significantly mitigated in hepatocytes neighboring cholangiocytes (*Spp1*,* C6*,* Cdh1*, and *Cd74*) (Fig. 8C), demonstrating that H19 deletion-mediated transcriptional recovery is spatially restricted to periductal hepatocytes.

## Discussion

A major gap in understanding PSC lies in elucidating how the disease progresses at the spatial and single-cell levels, and how different interventions influence these processes. While previous research has established that H19 exacerbates PSC disease progression, its in vivo effects at cellular and spatial transcriptomic resolution remain poorly understood [12, 14–19]. In this study, supported by recent findings from *Andrews*, et al., we utilized an integrative snRNAseq, spatial transcriptomic, and machine learning approach to elucidate cell type-specific and spatially restricted alterations in PSC [37]. Furthermore, we demonstrate how H19 deletion mitigates these pathogenic transcriptional states and shifts disease trajectories at both single-cell and spatial levels.

Our analysis identified a disease-specific cholangiocyte subcluster characterized by high expression of *Csmd1*,* Ccdc141*, and *Lama4*, which was significantly reduced following *H19* deletion (Supp. Fig. 1). *Lama4*, a laminin family extracellular-matrix glycoprotein, provides a mechanistic link between *H19* deletion and the attenuation of fibrosis in cholestatic liver disease. Using multimodal approaches, we identified six genes that robustly distinguished healthy from diseased cholangiocytes across scRNA-seq and combined sc/snRNA-seq datasets. Notably, the simplest model, logistic regression, achieved strong performance, suggesting these genes define a relatively linear health vs. disease axis. Several disease-specific genes (*Anxa3*, *Slco3a1*, and *Cftr*) were significantly reduced following H19 deletion (adj. *p* < 0.001, DKO vs. Mdr2KO) (Fig. 4C). *Anxa3* encodes Annexin A3, a calcium-dependent phospholipid-binding protein elevated in liver cancers and explored as a diagnostic and therapeutic target [38, 39]. Its increase in PBC and PSC patients is likely linked to cholangiocyte proliferation tied to ductular reaction. *Slco3a1* (OATP3A1), a sodium-independent organic anion transporter linked to Crohn’s disease and NF-κB activation [40], also mediates bile-acid efflux and has been proposed as a protective adaptive response in cholestasis [41]. Although *Slco3a1* expression was reduced in DKO vs. Mdr2KO (adj. *p* < 0.001), it was unchanged in H19KO vs. WT (*p* > 0.05), indicating that its down-regulation likely reflects reduced cholestasis rather than direct H19 regulation. The increase in these ion transporters in cholestatic liver disease may be a compensatory mechanism to aid in fluid secretion into the canaliculi to promote improved bile flow and eliminate blockages.

Two healthy cholangiocyte markers, Clu and Spp1, exhibited striking shifts: both were downregulated in diseased cholangiocytes and ectopically expressed in hepatocytes in diseased livers. Reduced level of circulating *CLU* protein (clusterin) has been associated with worse outcomes in biliary atresia [42]. In our model, H19 deletion mitigated the ectopic increase in hepatocyte *Spp1* expression (*p* < 0.05). In pancreatic ductal adenocarcinoma, Spp1 signaling sustains mesenchymal transition, while its inhibition decreases tumor burden and improves survival [43]. Cholangiocytes have the highest expression of *Spp1* in healthy livers [44]. We find that during cholestatic injury, Spp1 signaling is elevated but primarily originates from disease-associated hepatocytes. RT-qPCR revealed that the total levels of *Clu* and *Spp1* were significantly increased in Mdr2KO mouse livers (Supp. Fig. 7). Thus, suppressing hepatocyte-derived Spp1 may limit HSC-to-myofibroblast transition while sparing normal cholangiocyte Spp1 activity.

SnRNA-seq analysis revealed distinct cell-type–specific alterations driven by disease and mitigated by H19 deletion. Diseased hepatocytes showed ectopic expression of genes normally restricted to other healthy cell types, *Spp1* and *Clu* (cholangiocytes) and *Cd74* (antigen-presenting cells), which was reversed toward to WT levels following H19 deletion (*Clu* and *Spp1* significantly increased in Mdr2KO vs. WT, and decreased in DKO vs. Mdr2KO; adj. *p* < 0.001) (Fig. 6, Supp. Fig. 6). Consistent with prior reports, hepatic *Cd74* up-regulation has been linked to *Ikbkb* loss; accordingly, Mdr2KO hepatocytes showed reduced *Ikbkb* expression relative to WT (adj. *p* < 0.001) [45]. Both *Cd74* and *Ikbkb* changes were reversed in DKO hepatocytes compared to Mdr2KO (adj. *p* < 0.001) (Supp. Table 3).

The predictive capabilities exhibited by these cell-type-specific models may aid in future mechanistic studies by prioritizing therapeutic targets at cellular resolution. Furthermore, as spatial and single-nucleus profiling are increasingly applied to clinical liver biopsies, incorporating these predictive signatures could improve prognostic capacity regarding time to graft failure or the risk of PSC recurrence following liver transplantation. However, future longitudinal studies incorporating these modeling measurements are needed to fully evaluate their clinical predictive value.

We also identified several cell type–specific differentially expressed genes associated with by *H19*. Predictive models distinguishing *H19* WT from *H19* knockout cells demonstrated strong performance across all cell types, confirming robust, cell type–specific transcriptional signatures. In hepatocytes, Spp1 and Cd74, both disease-associated genes, were restored towards WT levels with H19 deletion. The APP-CD74 signaling axis has previously been shown to promote fibrosis in the kidney [46], highlighting a potential conserved fibrogenic mechanism. Additional hepatocyte-specific H19-responsive genes included lipid metabolism regulators, Cers6, Ces1c, *Apob*, and *Ahsg*, all of which were reduced following H19 deletion. Elevated *Ahsg* expression has been linked to tumor proliferation [47]. In cholangiocytes, H19 deletion-associated genes included *Frmd4b*, *Chka*, *Csmd1*, *Gnas*, and *Agmo*. Although increased *Csmd1* expression has been associated with HCC [48], other studies suggest it may function as tumor suppressor depending on context [49, 50].

GeoMx spatial transcriptomics demonstrated that the amelioration of hepatocyte disease-specific gene expression resulting from H19 deletion is confined to hepatocytes adjacent to bile ducts. This aligns with prior reports showing that cholangiocytes are the predominant source of H19 in cholestatic injury and that H19 is transferred to neighboring cell types through extracellular vesicles [14, 15, 17].

Taken together, these findings demonstrate that H19 deletion drives cell-type– and spatially specific transcriptomic remodeling, restoring healthy gene-expression programs across multiple hepatic lineages and mitigating PSC progression in both mouse and human datasets.

### Short comings

While we successfully validated our disease-associated differential gene-expression patterns and predictive modeling results using an independent human dataset, we were unable to validate the H19-deletion-specific findings due to the lack of comparable datasets. Additionally, the use of one mouse per genotype may limit the generalizability of individual genotype-level comparisons. Moreover, the sequencing depth in both our mouse and publicly available human snRNA-seq datasets was insufficient to reliably capture H19 expression, and H19 was not included in the GeoMx Whole Transcriptome Atlas (WTA) panel. Collectively, these constraints limited our ability to perform direct cross-species validation of H19-deletion effects at either the single-cell or spatial resolution.

## Supplementary Information

Below is the link to the electronic supplementary material.

Supplementary Material 1

## Data Availability

All data and analysis methodology will be made available upon requests to the corresponding author. snRNAseq and spatial transcriptomic data has been uploaded to GEO, series GSE311100.

## Abbreviations

AF: Alexa fluor
aSMA: alpha Smooth Muscle Actin
AUC: Area under the curveDEGs–differentially expressed genes
DGE: Differential gene expression
DKO: Mdr2KOH19–^/^–double knockout
DSP: Digital spatial profiler
FN1: Fibronectin
HCC: Hepatocellular carcinoma
H19: long non–coding RNA H19
H19KO: H19 knockout
LASSO: Least Absolute Shrinkage and Selection Operator
Mdr2: Multidrug resistance 2
MLM: Machine learning models
NDD: Neurologically deceased donor
PBC: Primary biliary cholangitis
PSC: Primary sclerosing cholangitis
RNA: Ribonucleic acid
ROC: Receiver operating characteristic
ROI: Regions of interest
RT: qPCR–real–time quantitative polymerase chain reaction
scRNAseq: Single cell RNA sequencing
snRNAseq: Single nuclear RNA sequencing
SPP1: Secreted phosphoprotein 1
WT: Wild type
WTA: Whole transcriptome atlas
