# Supplementary material for: Decoding the role of H19 in cholestatic liver injury using snRNA-seq, spatial transcriptomics, and machine learning-based disease prediction
Source: Cell Biosci. 2026 May 29;16:84. doi: 10.1186/s13578-026-01590-3 (PMC13425977; doi:10.1186/s13578-026-01590-3)

**Supplemental Methods:**

**Sequence mapping and filtering**

Original sequencing procedures were performed by SingulOmics. Nuclei from the four frozen mouse liver tissue samples were isolated. 3’ single cell gene expression libraries (Next GEM v3.1) were constructed using the 10x Genomics Chromium system. The libraries were then sequenced with ~200 million PE150 reads per sample on Illumina NovaSeq. After sequencing, clean reads were mapped into count data with mouse reference genome mm10 using Cell Ranger (v6.0.1). Filters of UMI>1200 and mitochondria < 10% were applied. Introns were included in the analysis. Samples were aggregated, and the gene-barcode matrices provided by SingulOmics were used for downstream analysis. The R package SoupX (version 1.6.2) was used to remove ambient RNA contamination(21). Endothelial-specific genes ***Ptprb, Pecam1, Stab2,*** *and* ***Eng*** were used as reference markers to assess ambient RNA contamination, based on their high expression specificity and tight clustering within endothelial cell populations in our mouse dataset (21). In the publicly available dataset GSE243981, raw reads were quality-controlled, mapped, and unique molecular identifiers (UMIs) quantified using Cell Ranger (v3). Viable cells were identified using EmptyDrops (v1.2.0), and ambient RNA contamination was corrected using SoupX (v1.2.2), as specified in the series matrix metadata(21, 22). For our analysis, we included scRNA-seq and snRNA-seq data from all primary biliary cholangitis (PBC) patients, six randomly selected primary sclerosing cholangitis (PSC) patients (GSM7883933, GSM7883934, GSM7883935, GSM7883936, GSM7883937, GSM7883938), and six randomly selected control patients (GSM7802679, GSM7802683, GSM7802688, GSM7802692, GSM7802693, GSM7802704). During our sample processing and analysis, a stricter < 2.5% mitochondrial genes filter was used. These data were used to evaluate the translational applicability of our mouse-derived gene prediction models in human liver disease samples.

**Gene selection for machine learning models**

DEGs were identified on a cell type basis (e.g., comparing only cholangiocytes to cholangiocytes), between healthy (WT and H19KO) and diseased (Mdr2KO and DKO) mice for disease prediction. Likewise, for H19 deletion associated gene prediction we compared H19-intact mice (WT and Mdr2KO) versus H19 knockout mice (H19KO and DKO). LASSO regression was run on all genes using Pearson residuals from Seurat's SCTransform. Final gene selection was performed using a consensus approach integrating statistical and biological criteria. Genes were required to rank highly in both the DEG analysis (by adjusted p-value) and LASSO regression (by coefficient magnitude). From this overlapping set, final candidates were selected based on biological plausibility within the cell type context, for example, ion transporters in cholangiocytes given their known role in ductal fluid secretion and relevance to the cholestatic phenotype of the Mdr2KO model.

**Neural Network Model**

A multilayer perceptron (MLP) classifier was implemented using the Keras API (R package keras v2.15.0) with a TensorFlow backend (R package tensorflow v2.16.0), interfaced via the reticulate package (v1.42.0) in R (v4.4.1)(34, 35). The network architecture consisted of two fully connected hidden layers with 16 and 8 units, respectively, each incorporating L2 regularization (λ = 0.01), batch normalization, and rectified linear unit (ReLU) activation functions. Dropout regularization (rate = 0.5) was applied following the first hidden layer to reduce overfitting. The output layer comprised a single neuron with a sigmoid activation function for binary classification. The model was trained using the Adam optimizer (learning rate = 0.0005) and binary cross-entropy loss function. Predictive performance was assessed using classification accuracy and the area under the receiver operating characteristic curve (ROC-AUC).

**XGBoost Model**

An XGBoost classifier was trained using a structured feature matrix with binary labels (0 = Healthy, 1 = Diseased). A grid search was conducted over hyperparameters including learning rate (η = 0.01, 0.05), tree depth (2, 3, 6), minimum child weight (2, 5, 10), and gamma (1, 2). Fixed parameters included a logistic objective, AUC evaluation metric, subsample ratio of 0.6, and L1/L2 regularization (α = 1, λ = 2). Five-fold cross-validation with early stopping (3 rounds) was used to select the best model based on mean AUC and stability (standard deviation < 0.02).

**Random Forest Model**

A random forest classifier was trained using the caret package in R(36, 37). A repeated 5-fold cross-validation (5 repeats) was used for model tuning. A grid search was performed over the number of variables randomly sampled at each split (mtry = 1 to 6). The model was trained with 500 trees, and accuracy was used as the tuning metric. The final model was selected based on cross-validated performance.

**Logistic Regression Model**

A logistic regression model was fitted using the training subset. The binary outcome variable was encoded as 1 (Diseased) and 0 (Healthy). The model was trained using the glm function in R with a binomial family and logit link. Probabilities were predicted on the test set, and model performance was evaluated using ROC-AUC.

**GeoMx® Digital Spatial Profiler**

Spatial transcriptomics was performed on liver sections from eight age-matched, 6-month-old female mice (four per genotype) across four slides using NanoString’s Whole Transcriptome Atlas (WTA) probe set and the GeoMx® Digital Spatial Profiler (DSP). Region-of-interest (ROI) selection was guided by three fluorescent morphology markers: AF488-conjugated anti–α-smooth muscle actin (αSMA), AF594-conjugated anti-CD68, and AF647-conjugated anti-EpCAM, along with the nuclear dye Syto 83, enabling identification of hepatocyte-, macrophage-, and bile duct–containing regions. Tissue preparation and processing were performed following the manufacturer’s protocol, GeoMx DSP Manual Slide Preparation: RNA FFPE (MAN-10150-02).

**RNA Isolation and RT-qPCR**

Total RNA was isolated from mouse liver tissue (5 male WT, 3 female WT, 5 male Mdr2KO, 3 female Mdr2KO) and human liver tissue (4 male healthy, 2 female healthy, 4 male PSC, 4 female PSC) using TRIzol Reagent (Thermo Fisher Scientific, Waltham, MA, USA) following the manufacturer's protocol. RNA was reverse transcribed into first-strand complementary DNA (cDNA) using the High-Capacity cDNA Reverse Transcription Kit (Life Technologies, Thermo Fisher Scientific, Waltham, MA, USA). Quantitative real-time PCR (qPCR) was performed using iQ SYBR Green Supermix (Bio-Rad) with glyceraldehyde 3-phosphate dehydrogenase (GAPDH) as an internal control. Primer sequences are listed in Supplemental Table 4. Gene expression was quantified using the ΔΔCt method. Statistical analysis was performed using two-way ANOVA (genotype × sex for mouse; disease status × sex for human). Where residuals violated normality (Shapiro-Wilk test, p < 0.05), rank-transformed ANOVA (Type III sum of squares) was applied. Normality and homogeneity of variance were assessed using Shapiro-Wilk and Levene's tests, respectively, to select post-hoc comparisons: Tukey's HSD (normal, equal variance), Games-Howell (normal, unequal variance), or Mann-Whitney U with Holm's step-down correction (non-normal). Significance was set at α = 0.05.

**Supplemental Tables:**

**Supp Table 1. Hepatocyte H19 expression associated genes that are also significantly associated with Mdr2KO and H19 deletion amelioration**

|  | **Mdr2KO vs DKO** | | | | | **Mdr2KO vs WT** | | | | | |
| --- | --- | --- | --- | --- | --- | --- | --- | --- | --- | --- | --- |
| **Gene** | **p_val** | **Log2FC** | **Pct.1** | **Pct.2** | **FDR** | **p_val** | **Log2FC** | **Pct.1** | **Pct.2** | **FDR** |  |
| Spp1 | 8.19E-138 | 1.595 | 0.452 | 0.215 | 6.14E-135 | 1E-323 | 5.564 | 0.452 | 0.039 | 7.26E-322 |  |
| Cd74 | 1.06E-102 | 1.325 | 0.383 | 0.185 | 2.82E-100 | 1E-323 | 3.433 | 0.383 | 0.094 | 7.25E-322 |  |

**Supp Table 2. Concordance of cholangiocyte top H19 expression associated genes and cholestatic liver injury associated gene expression amelioration**

|  | **Mdr2KO vs DKO** | | | | | **Mdr2KOvsWT** | | | | | | |
| --- | --- | --- | --- | --- | --- | --- | --- | --- | --- | --- | --- | --- |
| **Gene** | **p_val** | **log2FC** | **pct.1** | **pct.2** | **FDR** | **p_val** | **log2FC** | **pct.1** | **pct.2** | | **FDR** |  |
| Frmd4b | 6.48E-127 | -3.661 | 0.012 | 0.301 | 1.49E-122 | 8.73E-18 | -3.375 | 0.012 | 0.237 | 2.01E-13 | |  |
| Gnas | 1.79E-114 | -7.969 | 0 | 0.23 | 4.13E-110 | 5.02E-24 | -7.904 | 0 | 0.194 | 1.15E-19 | |  |
| Csmd1 | 6.30E-111 | 3.591 | 0.359 | 0.054 | 1.45E-106 | 9.02E-14 | 5.598 | 0.359 | 0.032 | 2.07E-09 | |  |
| Agmo | 1.54E-98 | -3.414 | 0.024 | 0.285 | 3.55E-94 | 1.77E-49 | -4.943 | 0.024 | 0.581 | 4.08E-45 | |  |

**Supp Table 3. Hepatocytes CD74 and Ikbkb DGE analysis by MAST**

|  | **Mdr2KO vs DKO** | | | | | **Mdr2KO vs WT** | | | | |
| --- | --- | --- | --- | --- | --- | --- | --- | --- | --- | --- |
| **Gene** | **p_val** | **log2FC** | **pct.1** | **pct.2** | **FDR** | **p_val** | **log2FC** | **pct.1** | **pct.2** | **FDR** |
| Cd74 | 1.06E-102 | 1.325 | 0.383 | 0.185 | 2.82E-100 | 1E-323 | 3.433 | 0.383 | 0.094 | 7.26E-322 |
| Ikbkb | 6.01E-08 | -0.330 | 0.26 | 0.28 | 1.17E-07 | 1.55E-21 | -0.463 | 0.26 | 0.335 | 3.08E-21 |

**Supp Table 4. qPCR primer sequences**

| **Species** | **Gene** | **Forward** | **Reverse** |
| --- | --- | --- | --- |
| Human | SLCO3A1 | GACCAGCCTGCGGGTTTATC | TGTCCAGGTTACTTGTGTCAATG |
| Human | ANXA3 | TTAGCCCATCAGTGGATGCTG | CTGTGCATTTGACCTCTCAGT |
| Human | CSMD1 | CGGGTATCCGAACTATGCCAA | GCAAAGGTATGGAAGGACAACT |
| Human | HAMP | CTGACCAGTGGCTCTGTTTTC | GAAGTGGGTGTCTCGCCTC |
| Human | CDH1 | ATTTTTCCCTCGACACCCGAT | TCCCAGGCGTAGACCAAGA |
| Human | GAPDH | ACATCATCCCTGCCTCTACTGG | TCCGACGCCTGCTTCACC |
|  |  |  |  |
| Mouse | Slco3a1 | AGGTGTCCTGCTTCTCCAAC | GTCAACACGCTCACCAGGTAG |
| Mouse | Anxa3 | ATGGCCTCTATCTGGGTTGGA | CAAGTCCTCTGATCGCTTTCC |
| Mouse | Cftr | CTGAAAGCAGGTGGGATTCT | CACAATGAACACCAACTGAATG |
| Mouse | Csmd1 | AGACGGAGTGCATACCCCAT | GCAGGTGTAGACTAAAGTGTAGC |
| Mouse | Hamp1 | TTATTTATTCCTGCCCTCCC | TAAAATCGTCTTTATTTCAAGGTC |
| Mouse | Hamp2 | CCTATCTCCAGCAACAGATG | AACAGATACCACAGGAGGGT |
| Mouse | Cdh1 | CAGGTCTCCTCATGGCTTTGC | CTTCCGAAAAGAAGGCTGTCC |
| Mouse | Clu | AGCTCCAAGAACTGTCCACTC | TATGTGCTTCACTCCCTGGAC |
| Mouse | Cd74 | CCGCCTAGACAAGCTGACC | ACAGGTTTGGCAGATTTCGGA |
| Mouse | Spp1 | GCTTGGCTTATGGACTGAGGTC | CCTTAGACTCACCGCTCTTCATG |
| Mouse | Gapdh | GTCGTGGATCTGACGTGCC | GATGCCTGCTTCACCACCTT |

**Supplementary Figure Legends:**

**Supp Fig 1. Top marker genes for the cholangiocyte disease–associated cluster.**

(A) Volcano plot showing differentially expressed genes for subclustered cholangiocytes, highlighting the top markers distinguishing cluster 2 from all other cholangiocyte clusters. Violin plots showing (B) significant increase in selected markers in cholangiocyte subcluster 2, (C) expression level by genotype, and (D) significant reduction by H19 deletion (H19-intact (WT and Mdr2KO) versus H19-knockouts (H19KO and DKO)). Marker genes were identified using Seurat’s FindMarkers function, comparing cluster 2 against all remaining clusters. For volcano plot, statistical significance was assessed using MAST with Benjamini–Hochberg correction (adjusted p < 0.05; |log₂FC| > 0.5). For violin plots, significance for contrasts performed with SeuratExtend with Wilcoxon test.

**Supp Fig 2. Summary of CellChat-inferred cell–cell communication.**

(A) Circle plots showing the number and strength of inferred cell–cell interactions across Wild Type, H19KO, Mdr2KO, and DKO mice. Curve colors correspond to the originating (outgoing) signaling cell type, and curve thickness reflects the relative number of interactions.
(B) Bar charts summarizing total inferred interaction counts and overall interaction strength for each genotype, illustrating the comparative increases or decreases in communication intensity across samples.

**Supp Fig 3. H19 deletion attenuates collagen signaling.**

CellChatv2 collagen signaling pathway analysis. A) Scatter plots and B) circle plots showing significant alterations in both signaling sources and target (receivers) cell populations when comparing diseased and healthy mice. Line colors denote the ligand-expressing (source) cell type, and line width represents the relative interaction strength within the collagen signaling network.

**Supp Fig 4. H19 deletion attenuates FN1 (fibronectin) signaling.**

CellChat v2 analysis of the fibronectin (FN1) signaling pathway.
(A) Scatter plots and (B) circle plots illustrating significant changes in both signaling source and receiver cell populations when comparing diseased and healthy mice. Line colors denote the ligand-producing (source) cell type, and line width reflects the relative interaction strength within the FN1 signaling network.

**Supp Fig 5. H19 deletion attenuates laminin signaling.**
CellChat v2 analysis of the laminin signaling pathway.
(A) Scatter plots and (B) circle plots demonstrating significant changes in both ligand-producing (source) and receiving cell populations when comparing diseased and healthy mice. Line colors indicate the signaling source, and line width represents the relative interaction strength within the laminin network.

**Supp Fig 6. Dot and violin plots illustrating disease- and cell-specific expression switching of Spp1 and Clu.**(A) Dot plot and (B) violin plots showing differential expression of the indicated features across disease states and relevant cell populations. Statistical significance was determined using the Wilcoxon test implemented in SeuratExtend (*p < 0.05, *****p*** < 0.01, ******p*** < 0.001, *******p*** < 0.0001)

**Supp Fig. 7. qPCR validation of disease prediction modeling genes.**

Bar plots showing qPCR results for candidate disease prediction genes in mouse (A) and human (B) hepatic tissue. Gene expression was quantified using the ΔΔCt method and normalized to GAPDH. Data are presented as mean fold change (2⁻ΔΔCt) ± SEM, with individual biological replicates shown as points. Statistical analysis was performed using two-way ANOVA (genotype × sex for mouse; disease status × sex for human). Where residuals violated normality (Shapiro-Wilk test, p < 0.05), rank-transformed ANOVA (Type III) was applied. Normality and homogeneity of variance were assessed using Shapiro-Wilk and Levene's tests, respectively, to determine post-hoc testing: Tukey's HSD (normal, equal variance), Games-Howell (normal, unequal variance), or Mann-Whitney U with Holm's step-down correction (non-normal). *p < 0.05, **p < 0.01, ***p < 0.001.

**Supp Fig 8. Hepatocyte zonation markers**

Dot plot showing the expression patterns of key hepatocyte zonation marker genes across hepatocyte subclusters.

**Supp Fig. 9. APP signaling pathway alterations across genotypes**.

Predicted APP signaling–related cell–cell communication and ligand–receptor interactions stratified by genotype. Bar plots illustrate the relative contribution of individual ligand–receptor pairs to APP signaling. Circle plots depict the corresponding cell–cell communication patterns for each contributing ligand–receptor interaction within each genotype.

**Supp Fig. 10. Random forest–based hepatocyte gene model for disease prediction.**A) ROC-AUC plot showing AUC score for the six-gene prediction model shown in panel B. B) Bar plots showing random forest model variable importance, indicating Gm13775 as the most important in differentiating between healthy and diseased hepatocytes. C) Violin plots showing expression of genes in the prediction model split on (i) cell type and by (ii) disease state.

**Supp Fig. 11. Cell type specific random forest models for H19 expression prediction.**
We selected 11 top genes identified by LASSO Regression were used to generate several cell type specific random forest models closely related to H19 expression split by cell type: ROC-AUC plots for A) cholangiocytes, B) hepatocytes, C) Macrophages.

**Supplemental Figures:**






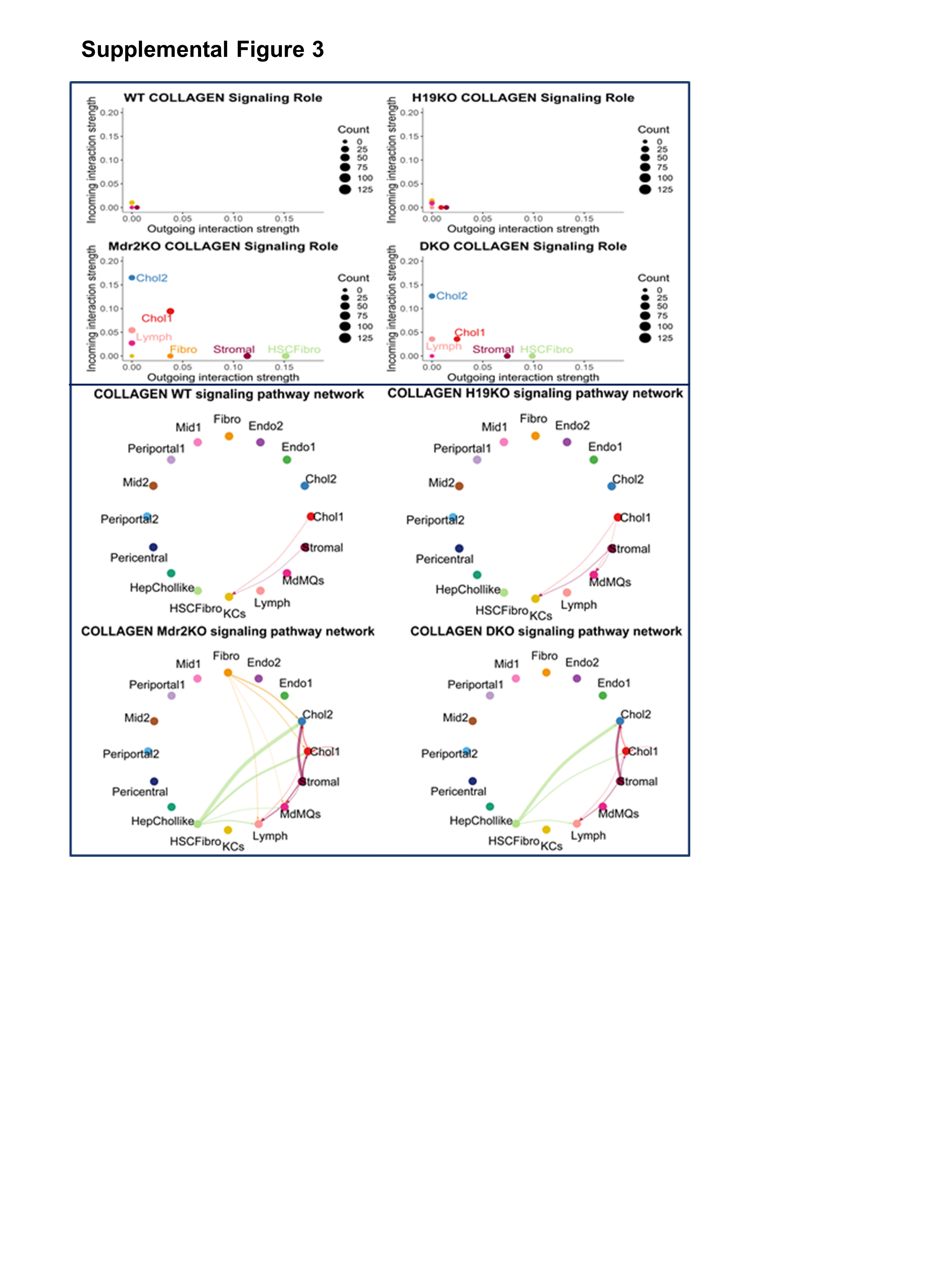


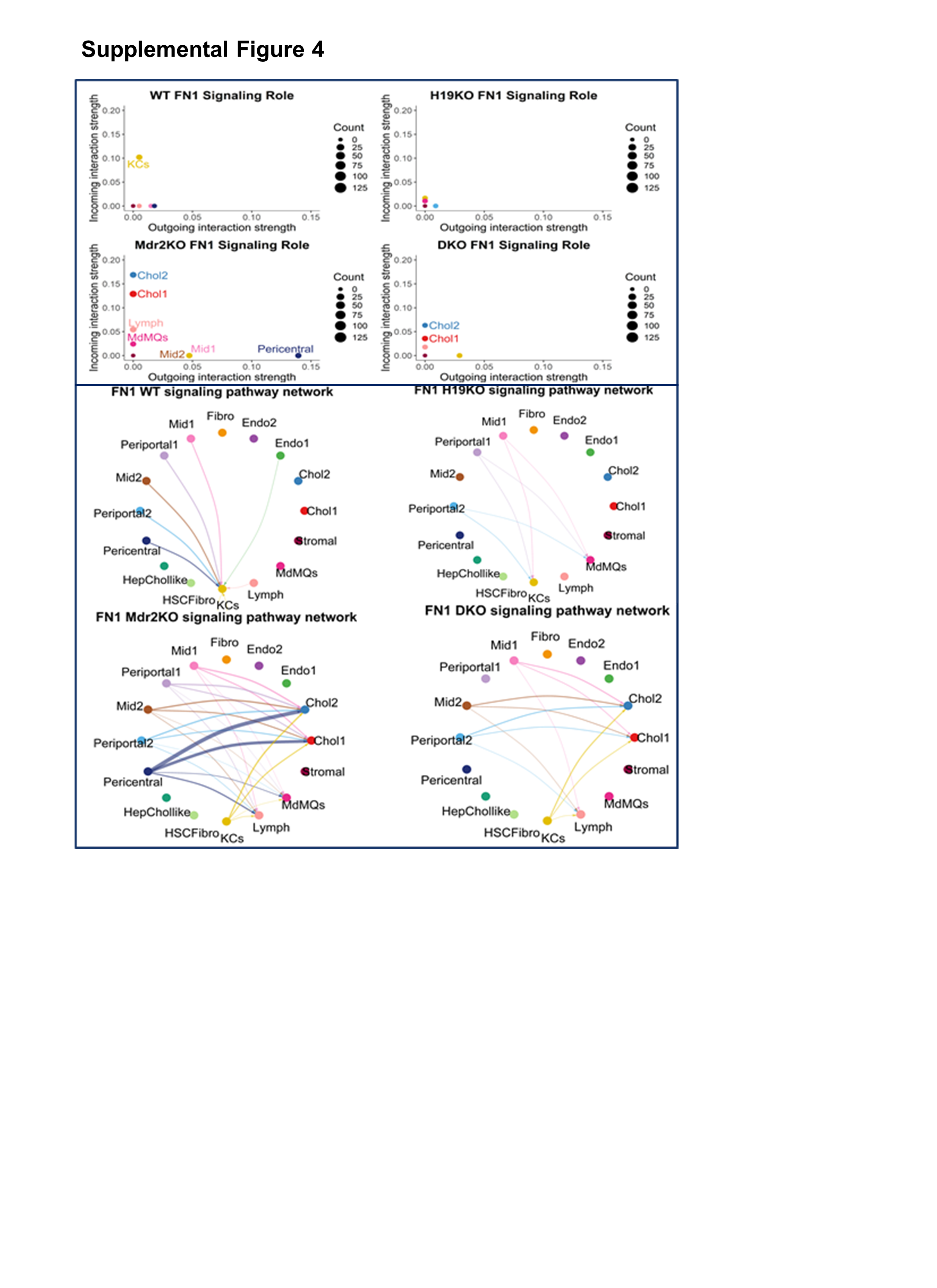


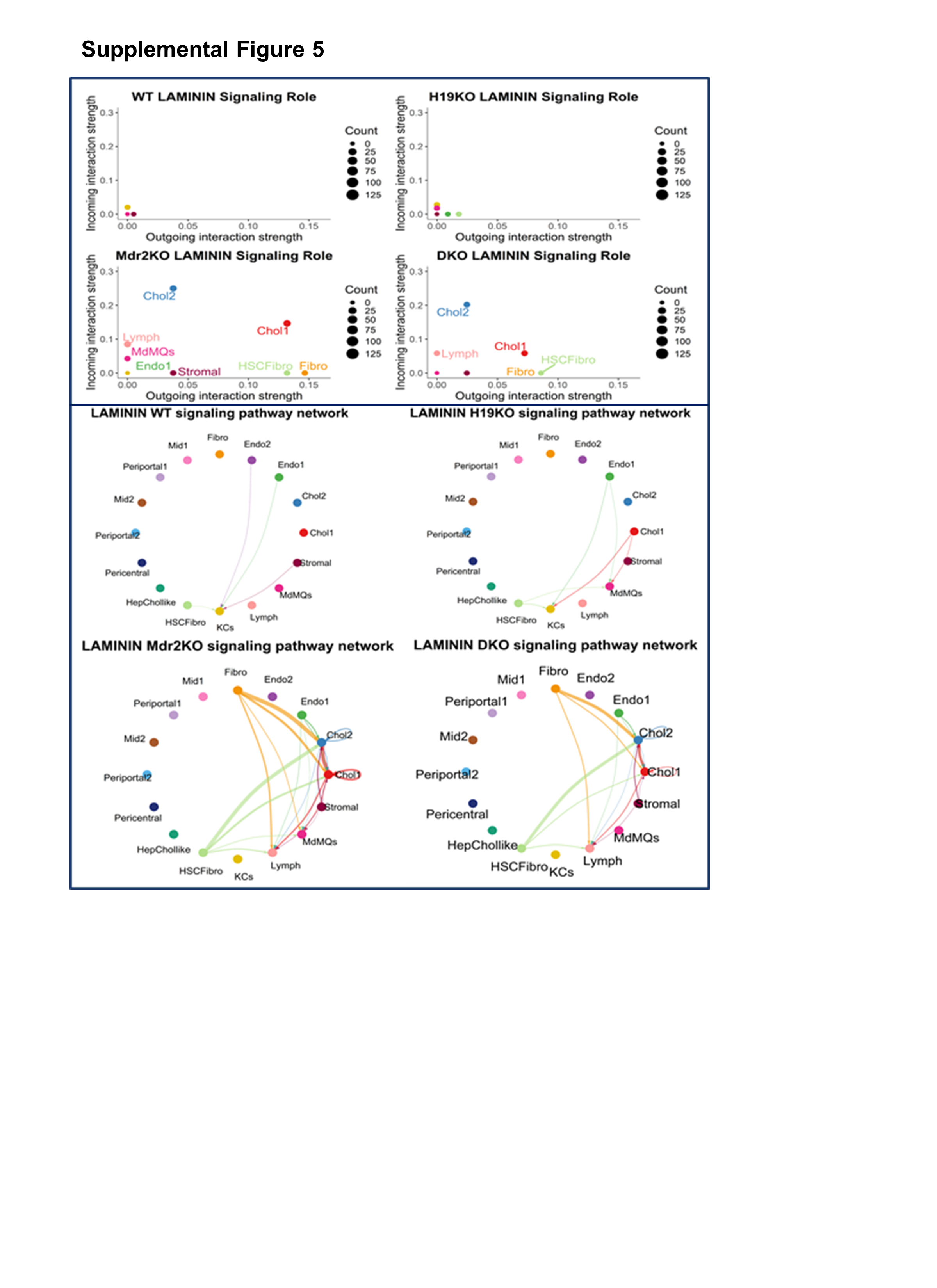


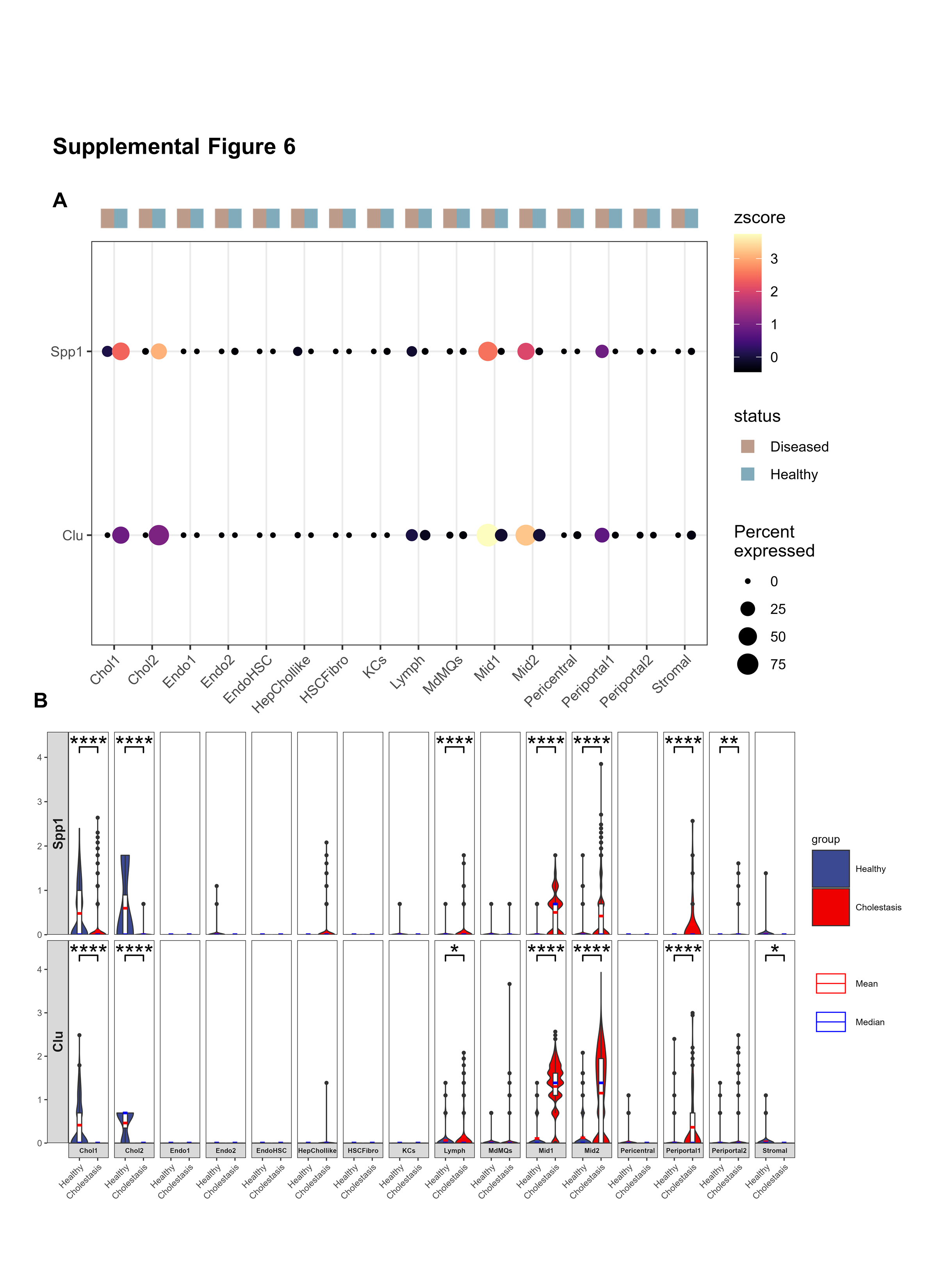

Supplement: Supplementary file 1 — Supplementary Material 1 [file 13578_2026_1590_MOESM1_ESM.docx]
